# Relationship between oral cancer and implants: clinical cases and systematic literature review

**DOI:** 10.4317/medoral.17223

**Published:** 2011-07-15

**Authors:** Enrique Jané-Salas, José López-López, Xavier Roselló-Llabrés, Oscar-Francisco Rodríguez-Argueta, Eduardo Chimenos-Küstner

**Affiliations:** 1 Doctor of Medicine and Surgery. Professor of Oral Medicine at the School of Dentistry, University of Barcelona; 2 Master’s degree in Dental Sciences. Department of Odontostomatology, University of Barcelona

## Abstract

The use of implants for oral rehabilitation of edentulous spaces has recently been on the increase, which has also led to an increase in complications such as peri-implant inflammation or periimplantitis. Chronic inflammation is a risk factor for developing oral squamous cell carcinoma (OSCC). Objectives: To review the literature of cases that associate implant placement with the development of oral cancer. Study design: We present two clinical cases and a systematic review of literature published on the relationship between oral cancer and implants. Results: We found 13 articles published between the years 1996 and 2009, referencing 18 cases in which the osseointegrated implants are associated with oral squamous cell carcinoma. Of those, 6 articles were excluded because they did not meet the inclusion criteria. Of the 18 cases reported, only 7 cases did not present a previous history of oral cancer or cancer in other parts of the body. Conclusions: Based on the review of these cases, a clear cause-effect relationship cannot be established, although it can be deduced that there is a possibility that implant treatment may constitute an irritant and/or inflammatory cofactor which contributes to the formation and/or development of OSCC.

** Key words:** Cancer, oral cancer, dental implants, oral squamous cell carcinoma, dental implants complications.

## Introduction

Between 3 and 5% of all malignant tumors are located in the head and neck region. Approximately half of these are located in the oral cavity, and oral squamous cell carcinoma (OSCC) accounts for roughly 90% of the total. It is defined as a malignant neoplasm which originates in the stratified epithelium, and is typically found in men over the age of 60, who have a habit of tobacco and alcohol use. However, this trend has been changing and is being observed more and more so in patients who are under the age of 40, even in children, adolescents, and in women who do not have any risk factors (1-2). It has also been associated with other less typical risk factors such as: the existence of nutritional deficiencies, exposure to ionizing radiation, immunosuppressant and irritant factors of dental and/or implant origin (3-5). 

Oral rehabilitation using osseointegrated dental implants has become one of the best options for the treatment of edentulous patients and is considered by some to be the only form of treatment (6). Due to the universality of the use of dental implants, the literature has also reported an increase in the number of complications associated with their use. Among such complications, the most frequent are the inflammatory processes that affect the bone and soft tissues, which are known as periimplantitis. Clinically, these conditions often occur with edema, erythema, hypertrophy and even ulcers of the soft tissues, sometimes presenting an appearance which may require a differential diagnosis with malignant lesions. 

To date, very few cases have been published on oral squamous cell carcinomas in proximity with osseointegrated dental implants, and even less on primary carcinomas in patients without a previous history of malignancy at the local or regional level (7,8). However, with the increase in the number of implants, we are likely to see an increase in the cases of oral squamous cell carcinoma. In this study, we present a review of the cases published in the literature on oral carcinomas associated with implants and we examine whether there is a direct relationship between the implants and the development of OSCC, evaluating the mechanism by which the implants may be considered risk factors. There is no data in the current literature that assess other possible causes, such as the galvanic effects that might arise from the transmembrane potential differences between the areas adjacent to the implants and the remaining mucosa; neither is there any data on the variables of decreased or increased bacterial colonization, or on the carcinogenic factors associated with the nitrosamines produced by Candida.

## Clinical Case No.1

The first case has to do with a 42-year-old male who has a clinical history of near morbid obesity (150 kg) and was treated with a dissociated diet for 9 years, which was unsuccessful. He was then surgically treated with a reduction gastroplasty in October 2008. The patient has no toxic habits at present (ex-smoker for 20 years) and has been under treatment for hypothyroidism with Eutirox®, one tablet daily for the past 15 years, now presenting normal thyroid hormone levels. The patient presented partial edentulism in both lower quadrants and was recommended implant placement in areas 36-37 and 46-47 as rehabilitation, which he accepted, and the implants were placed in September 2007.

**Figure 1:**
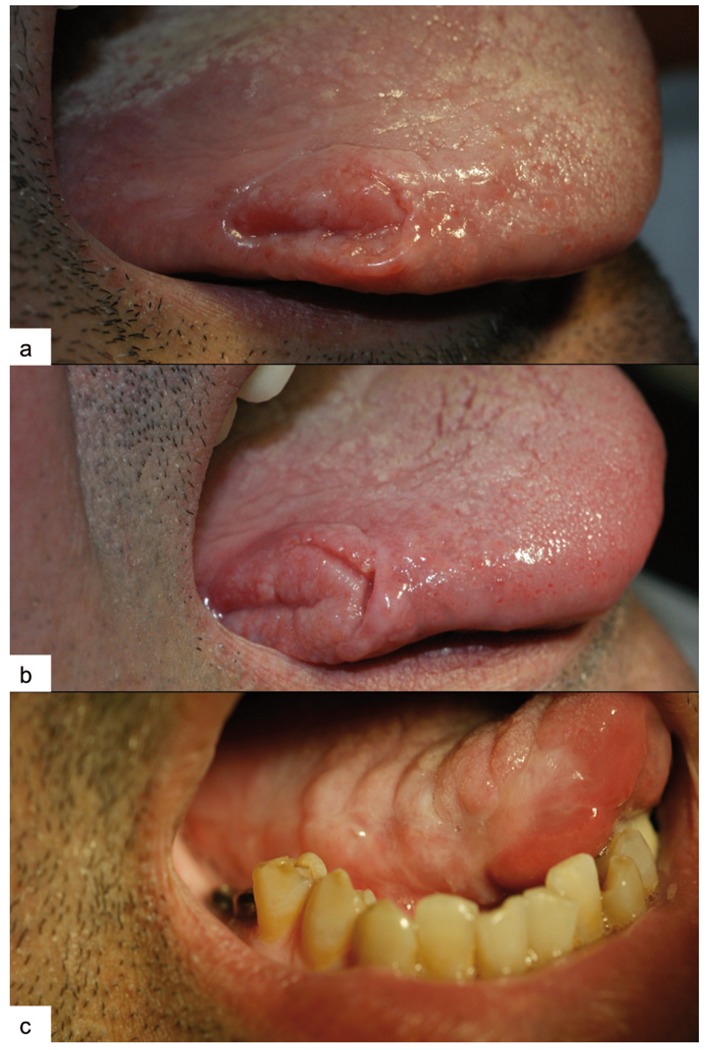
Clinical case no.1. A) Appearance of the lesion at the time of diagnosis. B) Above lesion after 2 months. C) Appearance of the tongue 2 months after treatment.

For various reasons, the patient did not undergo a prosthetic phase and did not come in for a check-up until December 29, 2008 (Fig. 1.A), presenting a non-painful, non-bleeding lesion on the right outer edge of the tongue, which the patient attributes to self-traumatism. Slightly hardened edges are observed upon palpation of the lesion, and examination of the lymph nodes in the neck and in the superclavicular area is negative. The patient refuses to undergo a biopsy. The patient is scheduled to be seen for a follow-up 10 days later (after eliminating possible traumatic factors); however, the patient does not show up for his appointment. We feel that it is important to see the patient again, and therefore urge him several times to come in for a follow-up. The patient does not come in for a visit until February 22, 2009 (Fig. 1.B), and upon observing the persistence of the lesion with negative cervical palpation, the patient is sent to the emergency room at the Head and Neck Functional Unit of Bellvitge University Hospital (Barcelona, Spain). There, the patient undergoes a biopsy and is diagnosed with OSCC, performing a right hemiglossectomy and functional homolateral lymph node drainage on March 25 (Fig. 1.C). The surgical piece revealed clean edges of the wound and absence of lymph node affectation. Therefore, the surgical team opted against additional treatment. At the 6-month follow-up (September 2009), there was no evidence of recurrence of the lesion and the PET-CT was negative. The next follow-up is scheduled in April 2010.

## Clinical Case No.2

**Figure 2:**
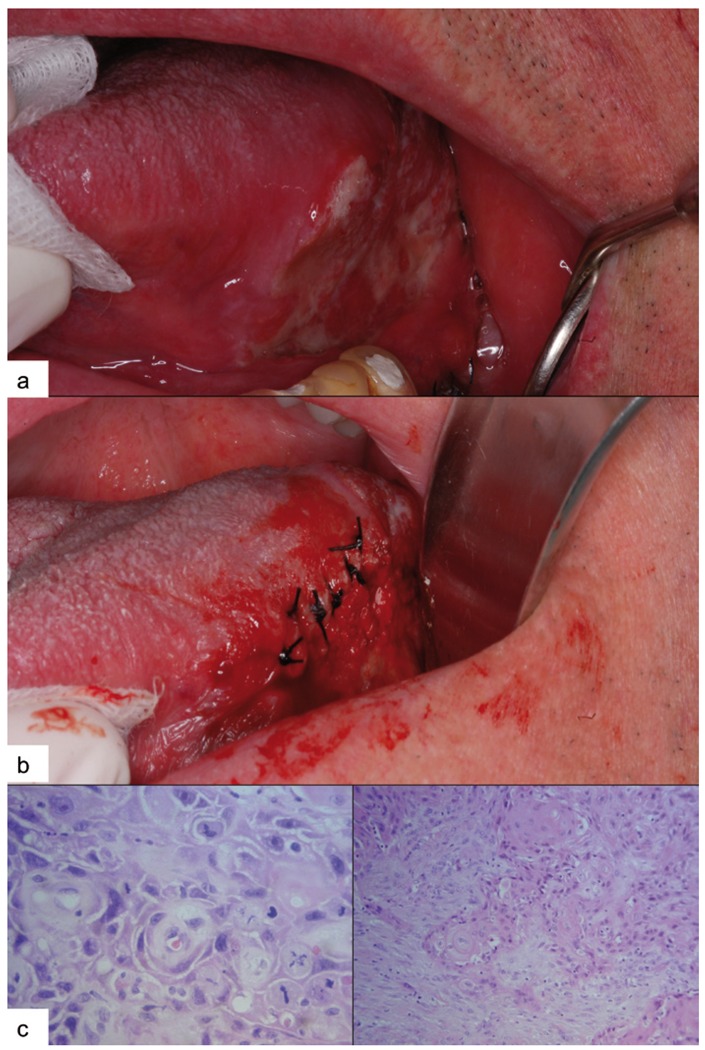
Clinical case no.2. A) Appearance of the lesion at the time of diagnosis. B) Biopsy of the suspected area. C) Histological image showing moderately differentiated OSCC.

The second case has to do with a 79-year-old male, without any relevant medical history, a non-smoker and non-drinker, wearing a full oral implant-supported denture for the past 9 years. In the upper arch, the rehabilitation contains 7 Branemark® implants and ranges from 1.7 to 2.7. In the lower arch, there are 5 Branemark® implants and the rehabilitation ranges from 3.5 to 4.5. The patient came in for an appointment on December 15, 2006, due to the presence of an ulcerated lesion on the middle third part of the left lateral edge of the tongue. Traumatic origin is suspected due to a fracture of the ceramic in the area. We proceed to polish the restoration and ask the patient to return for a follow-up appointment after 8 days. During the follow-up visit, an improvement of the lesion is noted and the patient agrees to have 3 additional implants placed in order to redo the lower rehabilitation. The treatment takes place on January 25, 2007, observing a small residual lesion in the ulcerated area. Upon removing the suture, we observe that the size of the lesion has increased (Fig. 2. A); therefore, an incisional biopsy is performed on February 8, 2007 (Fig. 2. B), sending the sample in for histopathological examination. The result reveals moderately differentiated oral squamous cell carcinoma (Fig. 2. C). At this time, the patient is referred to the Head and Neck Functional Unit in order to proceed with treatment, which consists of a hemiglossectomy and functional homolateral lymph node drainage. No recurrence of the lesion has been observed during the follow-up appointments to date.

 -Study design

We carried out a systematic review of the literature published in Medline (Pubmed) and in the SCIELO index, as well as in the COCHRANE database, using the terms: MeSH (Medical Subject Heading) “Dental Implants” and “Cancer”, and using the Boleean operator “AND”, in order to search for articles on the relationship between oral cancer and implants. As inclusion criteria, we considered articles which described cases of cancers that had developed subsequent to the placement of implants, without a prior history of oral cancer or cancer in any other part of the body, nor a history of any lesions classified as pre-malignant. Our studies did not include patients who had undergone prosthetic rehabilitation following treatment for cancer, nor any articles describing cases of patients who presented pre-malignant lesions, cancer in the mouth or in any other part of the body, prior to treatment with dental implants.

## Results

We found 13 articles published between the years 1996 and 2009, referencing 19 cases in which the osseointegrated implants are associated with oral squamous cell carcinoma ( Table 1). Seven cases of cancer (reported by six papers) presented a prior history of cancer in other regions, oral squamous cell carcinoma, pre-malignant lesions and a case of metastatic breast adenocarcinoma. Six cases of cancer (reported by five articles) did not present a prior history of squamous carcinoma, cancer in another region of the body or a pre-malignant lesion. And two articles presented patients with and without a prior history of cancer (three cases of cancer with previous history and three cases without previous history). 

Of the 19 cases reported, only 9 cases did not present a previous history of oral cancer, premalignant oral lesions or cancer in other parts of the body.

## Discussion

The mechanism by which osseointegrated dental implants could contribute to the development of oral squamous cell carcinoma is very debatable. Some authors argue that the placement of implants may contribute to the development of oral squamous cell carcinoma from the epithelium to the cancellous bone due to the loss of periodontal ligament (9).

**Table 1:**
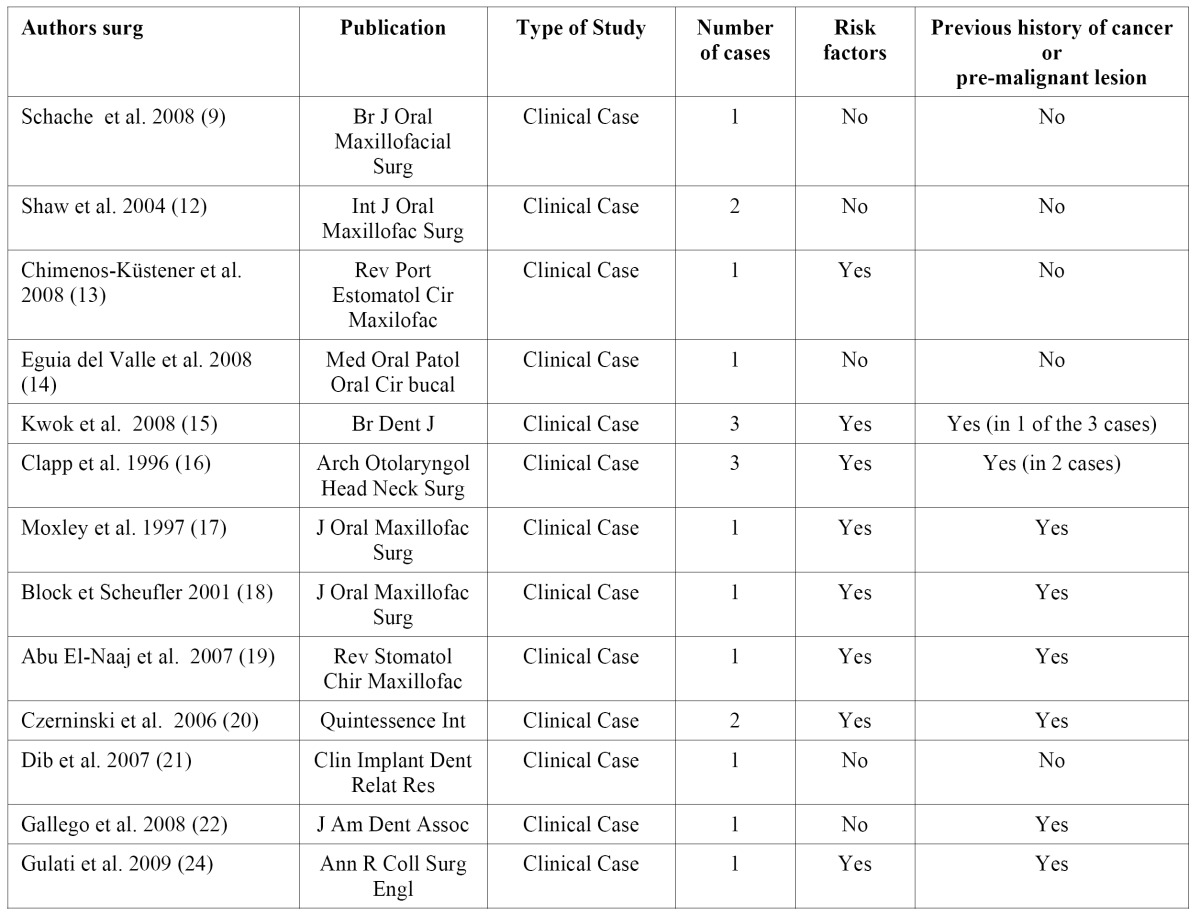
Summary of the articles reviewed. We present 19 cases of oral squamous cell carcinoma. 9 without prior history and 10 cases present previous history of squamous carcinoma, cancer in another region of the body or oral premalignant lesion.

The gingival attachment in implants is an area that experiences constant inflammation, which may affect the stability of the mucosa, and this inflammation may play an important role in the development of cancer due to the action of cytokine mediators such as: prostaglandins, interleukin-1, interleukin-6 and tumor necrosis factor. Additional risk factors may also be added, such as: alcohol and tobacco consumption, irritating factors such as an improperly fitted prosthesis or poor oral hygiene (10-11). In the literature reviewed, the vast majority of cases referred were of patients with oral squamous carcinoma or with a history of cancer in other parts of the body prior to implant placement. 

Nutritional deficiencies may explain the rapid evolution of a lesion, such as we present in case study 1, given that gastroplasty reduces the area of absorption for some nutrients and vitamins at the stomach level, increases the gastrointestinal transit rate and also reduces the absorption of certain necessary elements - all of which are associated with the local irritation factor of implants, which could explain the process in a patient who does not have any toxic habits.

Of the 12 cases described, only 6 of the patients did not present a prior history of carcinomas or cancer. In 2004, Shaw et al. (12) presented 2 cases of squamous cell carcinoma: one in a 67-year-old male and another in a 64-year-old female patient, without a prior history of malignant lesions or risk factors. In both cases, the lesions appeared to be compatible with those associated with peri-implantitis. In 2008 Schache et al. (9) reported a case in a 77-year-old male with an exophytic lesion located in the left mandibular region, associated with implants that support a fixed denture which was placed 5 years ago. The patient has no history of cancer, peri-implantitis or inflammation of the mucosa, nor any known risk factors. In 2008, Chimenos-Küstner et al. (13) present a case of a 62-year-old female whose risk factors include moderate alcohol consumption and being an ex-smoker, without a prior history of cancer, presenting an exophytic lesion around implants placed in the lower incisor region. The histopathological examination revealed an invasive moderately differentiated squamous cell carcinoma, requiring resective surgery with safety margins, as well as a functional radical homolateral lymphadenectomy. In 2008, Eguia del Valle et al. (14) present a case of a 76-year-old male with a history of hypertension and hyperuricemia, who received 2 implants placed in the right hemimandible (4.5 and 4.6) approximately 5 years ago, subsequently developing an exophytic lesion adjacent to 4.6. The patient did not present risk factors and was seen regularly for check-ups. As a first treatment, a peri-implant curettage was performed and a biopsy was performed 15 days later. The histopathological examination revealed a well-differentiated squamous cell carcinoma. Excision of the tumor was then performed with safety margins, without lymph node involvement.

Of the other articles published, 2 present patients with and without a prior history of cancer and the following 6 had a history of oral cancer or cancer in other parts of the body, pre-malignant lesions or metastases. In 2008, Kwok et al. (15) presented 3 cases. The first case concerns a 62-year-old male with poorly adapted dentures, who had been treated with 8 implants and has a history of high consumption of tobacco and alcohol. Three months after treatment, an ulcer was found in the lower right premolar region, whose histopathological study revealed a well-differentiated carcinoma. The second patient is a 71-year old male whose risk factors include heavy alcohol consumption and being an ex-smoker. He was treated with 2 implants in the lower incisor region and 6 years later an ulcer appeared which ended up being a well-differentiated squamous cell carcinoma, which was subsequently treated with surgery. The third patient is a 67-year-old female with a history of 2 small carcinomas in the lateral edges of the tongue, which were removed in 2001 and 2004. She was an ex-smoker and consumes alcohol in moderation. A breast tumor had also been removed in 1992. In 2006, she developed a hyperplastic lesion in the lower lip and during surgery, a small area of granulation tissue was found around the lower left implant. A biopsy was performed, revealing an early stage of squamous cell carcinoma adjacent to the implant. The patient was thus treated with local excision of the carcinoma. In 1996, Clapp et al. (16) reported three cases, one of which did not have a history of risk factors. The second case presented moderate dysplasia of the oral mucosa and the third case presented a prior history of OSCC. In 1997, Moxley et al. (17) reported a case, without risk factors, although with a history of verrucous carcinoma in the molar area. In 2001, Block and Scheufler (18) reported a case of a patient who was an ex-smoker and had a history of verrucous carcinomas in different areas and at different times. In 2007, Abu El-Naaj et al. (19) presented a case of a patient who is a smoker and has a history of oral lichen planus, presenting an exophytic lesion around implants in the symphyseal region. In 2006, Czerninski et al. (20) presented 2 cases: a patient with a high consumption of tobacco and a history of oral lichen planus, and the other patient with a prior history of oral and colon cancer. In 2007, Dib et al. (21) presented a case of a woman with metastasis around osseointegrated implants produced by a breast adenocarcinoma, which were diagnosed at the same time. In 2008, Gallego et al. (22) presented a case of a patient without any risk factors, with a history of oral lichen planus, who subsequently presents a carcinoma in situ in another location. The patient is treated with a resection of the area and rehabilitated with implants; however, due to the recurrence of carcinoma, a mandibular resection next to the implants is performed. 

There is a case of osteosarcoma in the upper maxilla (23) associated with implant placement with the use of fill material at the level of the maxillary sinus, which opens another debate about the use of platelet-rich factors or plasma rich in growth factors (PRF, PRGF). 

The article by Gulati et al. (24) describes a 62-year-old patient who was diagnosed with a verrucous leukoplakia located in the mandibular gingiva, which upon biopsy proved to be an invasive carcinoma, requiring surgical treatment with microvascular fibula graft. The patient had undergone posterior implant treatment for the prosthetic rehabilitation of the back part of his mouth, repeatedly suffering a peri-implantitis. Finally, a biopsy was performed in the inflamed area, which led to a diagnosis of oral squamous cell carcinoma. 

The role of osseointegrated implants in the formation of squamous cell carcinoma is not well-established, although the inflammation that occurs in the adjacent tissues may be an important factor which contributes to the development of this pathology.

Before an implant treatment, the patient’s risk factors must be established and an appropriate cost-benefit evaluation must be conducted for each patient.

In patients with risk factors, regular check-up should be performed in which a thorough examination of the oral cavity is performed, and in the case of a lesion that raises any questions, a biopsy and the subsequent histopathological examination should be performed in order to make a correct diagnosis as soon as possible.

The most frequent carcinoma associated with dental implants occurs in the form of peri-implantitis; that is why any of these symptoms requires thorough monitoring in order to carry out the final screening of carcinoma.

Therefore, we believe that from a medical standpoint, any implant worn over a denture should be able to be removed relatively comfortably in order to examine the peri-implant tissues and to monitor possible changes in this area.

## References

[1] Kademani D (2007). Oral cancer. Mayo Clin Proc.

[2] Kademani D, Bell RB, Schmidt BL, Blanchaert R, Fernandes R, Lambert P (2008). Oral and maxillofacial surgeons treating oral cancer: a preliminaryreport from the American Association of Oral and Maxillofacial Surgeons TaskForce on Oral Cancer. J Oral Maxillofac Surg.

[3] Warnakulasuriya KA, Ralhan R (2007). Clinical, pathological, cellular and molecular lesions caused by oral smokeless tobacco--a review. J Oral Pathol Med.

[4] Jane C, Nerurkar AV, Shirsat NV, Deshpande RB, Amrapurkar AD, Karjodkar FR (2006). Increased survivin expression in high-grade oral squamous cell carcinoma: a study in Indian tobacco chewers. J Oral Pathol Med.

[5] Guha N, Boffetta P, Wünsch Filho V, Eluf Neto J, Shangina O, Zaridze D (2007). Oral health and risk of squamouscell carcinoma of the head and neck and esophagus: results of two multicentriccase-control studies. Am J Epidemiol.

[6] Henry PJ (2005). Oral implant restoration for enhanced oral function. Clin Exp Pharmacol Physiol.

[7] Lemmerman KJ, Lemmerman NE (2005). Osseointegrated dental implants in private practice: a long-term case series study. J Periodontol.

[8] McDermott NE, Chuang SK, Woo VV, Dodson TB (2003). Complications of dental implants: identification, frequency, and associated risk factors. Int J Oral Maxillofac Implants.

[9] Schache A, Thavaraj S, Kalavrezos N (2008). Osseointegrated implants: a potential route of entry for squamous cell carcinoma of the mandible. Br J Oral Maxillofac Surg.

[10] Weitzman SA, Gordon LI (1990). Inflammation and cancer: role of phagocyte-generated oxidants in carcinogenesis. Blood.

[11] Jeng JH, Wang YJ, Chiang BL, Lee PH, Chan CP, Ho YS (2003). Roles of keratinocyte inflammation in oral cancer: regulating the prostaglandin E2, interleukin-6 and TNF-alpha production of oral epithelial cells by areca nut extract and arecoline. Carcinogenesis.

[12] Shaw R, Sutton D, Brown J, Cawood J (2004). Further malignancy in field change adjacent to osseointegrated implants. Int J Oral Maxillofac Surg.

[13] Chimenos-Küstner E, López-López J, Finestres-Zubeldia F (2008). Squamous carcinoma after dental Implants: A Clinical Case. Rev Port Estomatol Cir Maxilofac.

[14] Eguia del Valle A, Martínez-Conde Llamosas R, López Vicente J, Uribarri Etxebarria A, Aguirre Urizar JM (2008). Primary oral squamous cell carcinoma arisingaround dental osseointegrated implants mimicking peri-implantitis. Med Oral PatolOral Cir Bucal.

[15] Kwok J, Eyeson J, Thompson I, McGurk M (2008). Dental implants and squamous cell carcinoma in the at risk patient--report of three cases. Br Dent J.

[16] Clapp C, Wheeler JC, Martof AB, Levine PA (ArchOtolaryngol Head Neck Surg. 1996). Oral squamous cell carcinoma in association with dental osseointegrated implants. An unusual occurrence.

[17] Moxley JE, Stoelinga PJ, Blijdorp PA (1997). Squamous cell carcinoma associated with a mandibular staple implant. J Oral Maxillofac Surg.

[18] Block MS, Scheufler E (2001). Squamous cell carcinoma appearing as peri-implant bone loss: a case report. J Oral Maxillofac Surg.

[19] Abu El-Naaj I, Trost O, Tagger-Green N, Trouilloud P, Robe N, Malka G (2007). Peri-implantitis or squamous cell carcinoma?. Rev Stomatol Chir Maxillofac.

[20] Czerninski R, Kaplan I, Almoznino G, Maly A, Regev E (2006). Oral squamous cell carcinoma around dental implants. Quintessence Int.

[21] Dib LL, Soares AL, Sandoval RL, Nannmark U (2007). Breast metastasis around dental implants: a case report. Clin Implant Dent Relat Res.

[22] Gallego L, Junquera L, Baladrón J, Villarreal P (2008). Oral squamous cell carcinoma associated with symphyseal dental implants: an unusual case report. J Am DentAssoc.

[23] McGuff HS, Heim-Hall J, Holsinger FC, Jones AA, O’Dell DS, Hafemeister AC (2008). Maxillary osteosarcoma associated with a dental implant: report of a case andreview of the literature regarding implant-related sarcomas. J Am Dent Assoc.

[24] Gulati A, Puthussery FJ, Downie IP, Flood TR (2009). Squamous cell carcinoma presenting as peri-implantitis: a case report. Ann R Coll Surg Engl.

